# Systematic evaluation of the efficacy of treatments for cesarean scar pregnancy

**DOI:** 10.1186/s12958-024-01256-0

**Published:** 2024-07-18

**Authors:** Haiying Sun, Juan Wang, Peiying Fu, Ting Zhou, Ronghua Liu

**Affiliations:** 1 Department of Obstetrics and Gynecology, Tongji Hospital, Tongji Medical College, Huazhong University of Science and Technology, 1095 Jiefang Anv. Wuhan, Wuhan, Hubei 430030 P.R. China; 2 Department of Obstetrics and Gynecology, Liyuan Hospital, Tongji Medical College, Huazhong University of Science and Technology, Wuhan, P.R. China

**Keywords:** Cesarean scar pregnancy, Dilatation and curettage, Hysteroscopy, Laparoscopy, Laparotomy

## Abstract

**Study objective:**

Cesarean scar pregnancy (CSP) is a type of ectopic pregnancy associated with severe complications, including significant hemorrhage, the potential need for hysterectomy, and life-threatening risks. Currently, two classification methods exist for CSP: Vial (type I^a^ and II^a^) and Chinese Expert’s Consensus (type I^b^, type II^b^, and type III^b^). However, these methods have limitations in guiding the selection of appropriate treatment plans for CSP. The purpose of this study was to systematically evaluate the effectiveness of various treatments for CSP within our clinic.

**Method:**

Our study included 906 patients with CSP from January 2013 to December 2018. The chi-squared test and logistic analysis were used to compare the clinical characteristics. The median and interquartile range (IQR) was calculated. We also analyzed whether preoperative application of methotrexate (MTX) could improve surgical outcomes and the relevant characteristics of misdiagnosed CSP patients.

**Results:**

There was a significant difference in gestational age, gestational sac diameter, gestational sac width, gestational sac area, remnant myometrial thickness, vaginal bleeding and preoperative hemoglobin levels (*p* < 0.001) but not in the incidence of residual tissue (*p* = 0.053). The other factors (intraoperative blood loss, hemoglobin decline, first hemoglobin after operation, total hospital stay, hospital stay after operation, transfusion and duration of catheter drain) were significantly different (*p* < 0.001). For type I^a^ and type I^b^ CSP, 39.3% and 40.2% of patients were treated with dilatation and curettage (D&E) under ultrasound, respectively. For type II^a^ and type III^b^ CSP, 29.9% and 62.7% of patients were treated with laparotomy, respectively. There were no differences in surgical methods, residual tissue and reoperation between the MTX and non-MTX groups (*p* = 0.20), but liver damage, hospital stay and pain perception were more remarkable in the MTX group. It is noteworthy that 14% of the patients were misdiagnosed with an intrauterine pregnancy. The incidence of misdiagnosis in type II^a^ CSP patients was higher than that in type I^a^ CSP patients (*p* < 0.001).

**Conclusion:**

For type I CSP patients, D&E under ultrasound or D&E under hysteroscopy should be recommended. For type III^b^ CSP patients, operative resection should be used. It is currently difficult to choose the appropriate treatment methods for type II^a^ or type II^b^ CSP patients.

## Introduction

CSP or cesarean scar ectopic pregnancy (CSEP) is an infrequent form of ectopic pregnancy characterized by the implantation of embryonic villi into the scar tissue resulting from a previous cesarean Sects. [1, 2]. The diagnosis of CSP/CSEP is typically established within the first 12 weeks of gestation, with an incidence estimated to range from approximately 1:2216 to 1:1800 [3]. The prevalence of CSP increases in tandem with the rise in cesarean section rates [4]. Failure to appropriately manage CSP may result in severe complications such as profuse hemorrhaging or uterine rupture, potentially necessitating hysterectomy. These outcomes pose significant risks to women’s reproductive health and even their lives, thus prompting considerable clinical focus on CSP [5].

Despite the existence of numerous management strategies for CSP, there remains a dearth of standardized global guidelines or a consensus on the optimal treatment approach [6]. Several treatment regimens have been suggested, encompassing both medical and surgical interventions [7, 8]. Medical approaches have involved the local or systemic administration of MTX, while uterine artery embolization (UAE) has been proposed as an adjunct treatment option. Surgical interventions have included high-intensity focused ultrasound (HIFU), dilation and curettage (D&C) guided by ultrasound, hysteroscopy, laparoscopy, laparotomy, transvaginal surgery, and various combinations of these procedures. However, the current lack of consensus in the field is primarily attributed to the limited amount of evidence available regarding the efficacy of each treatment modality [9]. Despite this, surgical interventions have demonstrated higher success rates compared to medical treatments, albeit with the drawback of increased blood loss due to hemorrhage [6]. While alternative treatment modalities exist, their application in the management of CSP is challenging due to the limited sample size and low quality of evidence, making it difficult to provide definitive guidance for care provision.

Currently, while an exact classification for CSP is lacking, the classification put forth by Vial is widely embraced by clinical practitioners [10]. In 2016, Chinese scholars introduced another CSP classification methodology [11]. Nevertheless, the precise guiding efficacy of these two classification methods in clinical settings remains largely unassessed by doctors. Additionally, certain doctors have put forward alternative CSP classification approaches [12]. The primary factors contributing to the confusion surrounding the aforementioned treatment plans may be attributed to the presence of multiple classification systems and the lack of research on the guiding role of these two classification methods in clinical practice. Conversely, the failure to promptly and accurately diagnose CSP can potentially lead to its misdiagnosis, thereby increasing the likelihood of adopting an inappropriate treatment plan and subsequently experiencing varying degrees of consequences.

The primary objective of this retrospective study was to conduct a systematic assessment of the effectiveness of treatments for CSP within our clinic, taking into account various factors. Specifically, our aims were to evaluate five commonly employed management strategies, compare clinical characteristics, intraoperative bleeding rate, intraoperative complication rate, misdiagnose, and main outcomes across these strategies, assess the proportions of treatment options utilized according to two existing CSP classification methods, and analyze the optimal treatment plan for different CSP subtypes.

## Method

### Patients

A total of 906 patients were retrospectively recruited from a database comprising 935 individuals with cesarean scar pregnancy who received treatment from 2013 to 2018. This trial was registered at chictr.org.cn as ChiCTR900024793 (https://www.chictr.org.cn/showproj.html?proj=41545). The study was approved by the Ethics Committee of Tongji Hospital, Tongji Medical College, Huazhong University of Science and Technology (No: TJ-IRB20191214). The inclusion criteria were as follows: (1) a history of cesarean section; (2) a history of amenorrhea and positivity for human chorionic gonadotrophin (HCG); (3) the presence or absence of vaginal bleeding; (4) the presence of the following CSP characteristics as determined by transvaginal ultrasonography: gestational sac (GS) located in the anterior wall of the uterus and the bladder; the muscle wall between the GS and the bladder was thin; no GS could be detected in the uterine cavity; color Doppler showed abundant peripheral blood flow with a high speed and low resistance spectrum; and the anterior wall of the uterus was sagittal and discontinuous; and (5) all therapeutic strategies were categorized into five distinct types: D&E under ultrasound (group 1), D&E under hysteroscopy (group 2), D&E under laparoscopy (group 3), or laparoscopy (group 4) and laparotomy (group 5). All operations were performed by gynecologists with 10 years of experience in surgical gynecology in our hospital. During surgery, the intraoperative volume of blood loss (ml) was recorded, and all tissues obtained were sent for pathological confirmation of pregnancy. Postoperative HCG values were tested on the second day after surgery.

### Clinical classification

At present, there are two common ways to classify CSP. The initial approach involves a binary classification that relies on the correlation between GS and scar.: type I^a^ (endogenic), when the GS grows inward toward the cervicoisthmic space; and type II^a^ (exogenic), when the GS grows outward to the bladder and abdominal wall. The second method is a three-way classification (Chinese Expert’s Consensus, 2016) [11]: type I^b^, when the GS grows into the cervicoisthmic space and the thickness of the myometrial wall was more than 3 mm; type II^b^, when the thickness of the myometrial wall is less than or equal to 3 mm; and type III^b^, when the GS shows deep invasion of the scar defect with a progression toward the bladder and abdominal cavity and the myometrium between the bladder and sac is less than 3 mm or missing entirely. Furthermore, abundant vascularization is present within the implantation site. This classification technique offers a higher level of granularity compared to the aforementioned method. In our study, the classification of CSP was determined through the consensus of Chinese experts.

### Treatments

#### Systemic methotrexate injection

In our study, patients with a HCG level greater than 5000 mIU/mL and abundant blood flow in the scar area underwent MTX treatment. The patients who received systemic injection were hemodynamically stable and did not have any contraindications for MTX, such as hepatic dysfunction, renal dysfunction, leukopenia, active peptic ulcer disease, or immunodeficiency disorders. Two injections of MTX (50 mg/m^2^) were administered on days 1 and 4. On the 7th day, the patient underwent the corresponding surgery.

### D&E under ultrasound

Curettage alone was conducted under ultrasound monitoring when it was ascertained that the majority of the GS resided within the uterine cavity, with no GS embedded in the myometrium and the scar thickness surpassing 2 mm. Before the procedure, ultrasound was used to verify the relationship between the GS and the scar in the lower segment of the uterus, the thickness of the scar, and the relationship between the uterus and the bladder. Our experience is that during the operation, the surgical instruments enter the uterine cavity from the posterior wall of the endocervix, and evacuation via negative pressure suction starts from the uterine cavity and gradually approaches the location of the GS and scar.

### D&E under hysteroscopy

The scar thickness of these patients exceeded 1 mm. The remaining indications for treatment were consistent with the selection criteria for D&E under ultrasound. First, we used hysteroscopy to observe the position of the GS and the relationship between the GS and the scar and compared it with the preoperative ultrasound results. After curettage, hysteroscopy was used to check for any residual pregnancy tissue and the presence of an incision diverticulum. If there was residual tissue in the diverticulum, tissue removal and electrocision were performed under direct vision. If there is bleeding at the attachment of the GS or diverticulum, electrocoagulation can be performed at the corresponding location.

### D&E under laparoscopy

This surgical method was adopted for patients with a scar thickness less than 10 mm or an exogenic CSP with abundant blood vessels. Laparoscopy can effectively evaluate the specific condition of the CSP site and whether blood vessels are abundant in the lower uterine incision. If there is excessive bleeding during curettage, timely and effective treatment can be performed quickly under laparoscopy.

### Laparoscopy and laparotomy

Laparoscopic or open surgery was chosen based on the patient’s ultrasound results and the doctor’s personal surgical skills. Most of these patients had type II^a^, type II^b^ or type III^b^ CSP and underwent the two kinds of surgical methods (laparoscopy and /or laparotomy). Based on the implantation of villi in the lower segment of the uterus and the abundance of blood vessels, surgeons decided whether to perform uterine artery or internal iliac artery occlusion first. The bladder was pushed aside, and the lower segment of the uterus was exposed. After complete removal of the pregnancy tissue and full excision of the CSP mass, the niche of the uterus was repaired. The surgical procedures of laparoscopy and laparotomy were performed according to our previously described method [13].

### Statistical analysis

IBM SPSS 26.0 statistical software was used to perform the statistical analysis. Quantitative data are typically characterized by mean ± standard deviation (SD). To compare two groups, two independent sample t-tests are employed, while analysis of variance is utilized for comparing multiple groups. Counting data, on the other hand, is described using frequency and composition ratio, and the comparison of compositions is carried out through the application of the chi-square test. In cases where the data fails to meet the requirements for the chi-square test, Fisher’s exact method is employed as an alternative. A statistically significant difference is indicated when the p-value is less than 0.05. The median and interquartile range (IQR) was calculated. All reported *p* values were two-sided, and we considered *p* < 0.05 to be the significance threshold.

## Results

### Clinical and sonographic characteristics of CSP patients

**Fig. 1:**
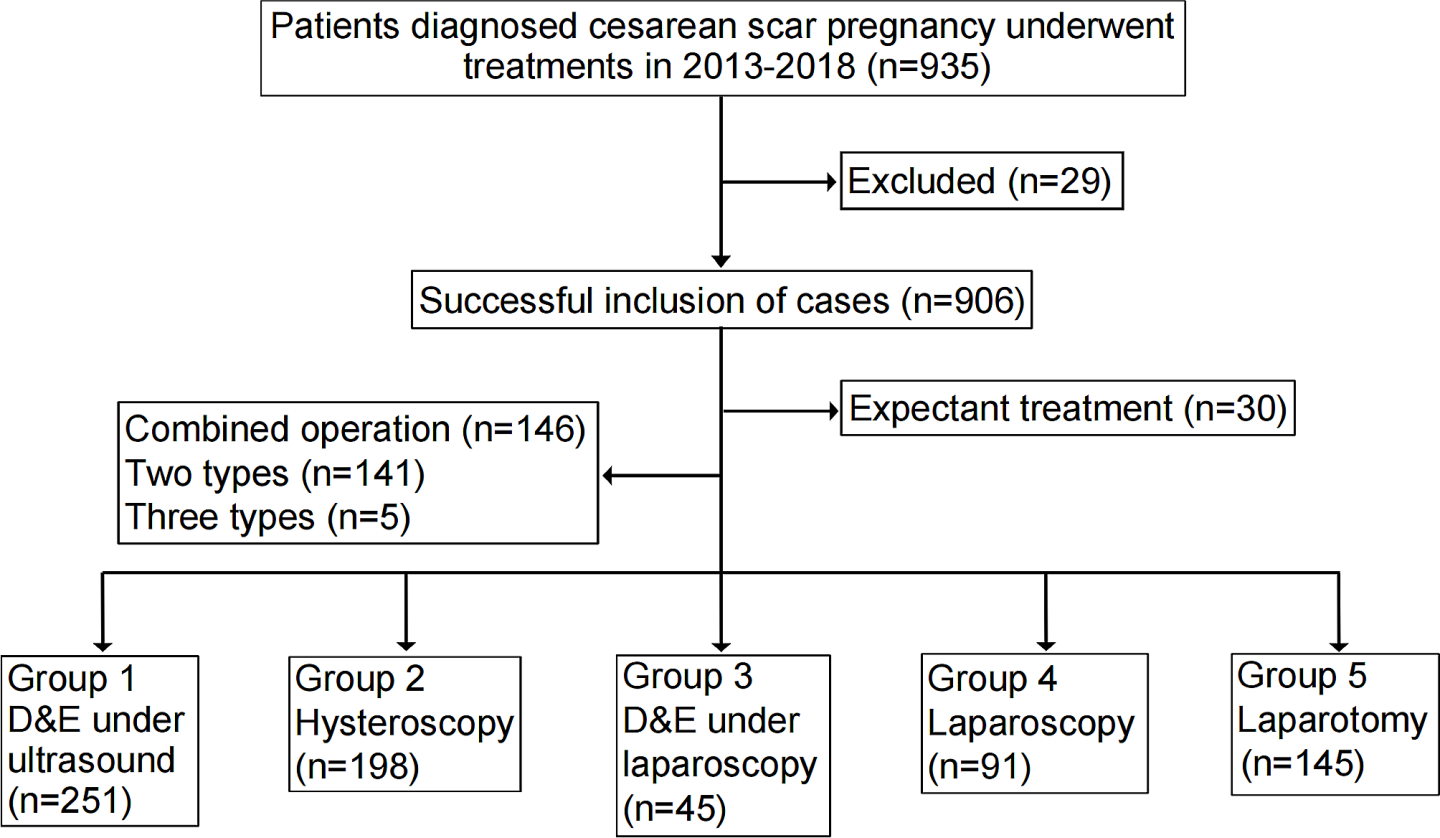
Patient and treatment characteristics

The data pertaining to 935 cases associated with CSP were extracted from our case database. Following verification, 29 cases of intrauterine pregnancy were identified and subsequently excluded. Among the remaining 906 CSP patients, 30 opted for expectant management to sustain their pregnancy. The majority of these patients exhibited a GS primarily situated within the uterine cavity. Due to the substantial rate of data loss observed in these patients, an analysis of subsequent pregnancy outcomes was not conducted. A combined operation was performed on a total of 146 patients, with 141 patients undergoing two types of operations and 5 patients undergoing three types of operations. The decision to employ a combination of treatment methods in these patients was influenced by their prior utilization of D&C under ultrasound or hysteroscopy. In cases where these procedures led to heightened bleeding during the operation, a transition to laparoscopic or open surgery may be warranted. The treatment regimen employed in this cohort of 730 patients consisted of a singular approach. Among these patients, there were 251 cases of D&E under ultrasound, 198 cases of hysteroscopy, 45 cases of D&E under laparoscopy, 91 cases of laparoscopy, and 145 cases of laparotomy (Fig. 1).

Notably, the majority of CSP patients were under the age of 35 (*p* = 0.004). Group 1 and group 2 comprised 39.3% and 46.7% of type Ia CSP patients, respectively. Group 4 and group 5 accounted for 20% and 29.9% of type II^a^ CSP patients, respectively, with a statistically significant difference (*p* < 0.001). The majority of patients diagnosed with CSP experienced amenorrhea for a duration of 43–64 days. Various ultrasonographic parameters, such as the diameter, width, and area of the gestational sac, as well as the residual myometrial thickness, demonstrated statistically significant variances (*p* < 0.001), along with the color doppler signal (*p* = 0.024), across the five groups (Table 1). However, there was no significant difference observed in fetal heartbeat among the groups (*p* = 0.18). The primary manifestation of CSP was vaginal bleeding, exhibiting notable variations across groups (*p* < 0.001). Conversely, no substantial disparity in the prevalence of abdominal pain was discernible among groups (*p* = 0.23). The proportion of individuals encountering vaginal bleeding was greater in groups 4 and 5 compared to the remaining groups. The preoperative hemoglobin levels of patients in the laparoscopy and laparotomy groups were lower than those in the other three groups (*p* < 0.001). All specimens were definitively identified as pregnant tissue.

**Table 1:** Univariate analysis of clinical and sonographic characteristics of women with cesarean scar pregnancy

Characteristic	Group 1	Group 2	Group 3	Group 4	Group 5	*p* value
Number of patients	251(%)	198(%)	45(%)	91(%)	145(%)	
Age (y)						0.004
<35	159 (63.3)	127 (64.1)	25 (55.6)	74 (81.3)	105 (72.4)	
≥35	92 (36.7)	71 (35.9)	20 (44.4)	17 (18.7)	40 (27.6)	
Urban residents						0.014
No	68 (27.3)	51 (25.8)	8 (17.8)	37 (40.7)	32 (22.1)	
Yes	181 (72.7)	147 (74.2)	37 (82.2)	54 (59.3)	113 (77.9)	
Type						< 0.001
Type I^a^ (*n* = 229)	90(39.3)	107(46.7)	21(9.2)	5(2.2)	6(2.6)	
Type II^a^ (*n* = 421)	86(20.4)	101(24.0)	24(5.7)	84(20.0)	126(29.9)	
Number of artificial abortions						0.43
0	53 (21.1)	28 (14.1)	9 (20.0)	22 (24.2)	29 (20.0)	
1	66 (26.3)	48 (24.2)	12 (26.7)	23 (25.3)	43 (29.7)	
2	63 (25.1)	54 (27.3)	11 (24.4)	26 (28.6)	41 (28.3)	
≥3	69 (27.5)	68 (34.3)	13 (28.9)	20 (22.0)	31 (21.4)	
Unknown	0 (0.0)	0 (0.0)	0 (0.0)	0 (0.0)	1 (0.7)	
Number of cesarean sections						0.36
1	186 (74.1)	150 (75.8)	31 (68.9)	59 (64.8)	101 (69.7)	
≥ 2	65 (25.9)	48 (24.2)	14 (31.1)	32 (35.2)	43 (29.7)	
Unknown	0 (0.0)	0 (0.0)	0 (0.0)	0 (0.0)	1 (0.7)	
Interval from last CS (y)						0.54
≤ 1	26 (10.4)	24 (12.1)	2 (4.4)	14 (15.4)	15 (10.3)	
2–4	81 (32.3)	63 (31.8)	13 (28.9)	35 (38.5)	44 (30.3)	
≥ 5	138 (55.0)	110 (55.6)	29 (64.4)	40 (44.0)	82 (56.6)	
Unknown	6 (2.4)	1 (0.5)	1 (2.2)	2 (2.2)	4 (2.8)	
Gestational age (d)						< 0.001
≤ 42	64 (25.5)	32 (16.2%)	12 (26.7%)	12 (13.2%)	23 (15.9%)	
43–63	152 (60.6)	138 (69.7%)	24 (53.3%)	53 (58.2%)	71 (49.0%)	
> 63	30 (12.0%)	27 (13.6%)	6 (13.3%)	25 (27.5%)	40 (27.6%)	
Unknown	5 (2.0%)	1 (0.5%)	3 (6.7%)	1 (1.1%)	11 (7.6%)	
Diameter of GS (cm), median (IQR)	2.7 (1.8, 3.8)	3.0 (2.0, 4.1)	3.0 (2.1, 4.0)	3.8 (2.6, 4.9)	4.3 (2.9, 5.8)	< 0.001
GS width (cm), median (IQR)	1.4 (0.9, 2.0)	1.5 (0.9, 2.1)	1.6 (1.2, 2.4)	2.6 (1.6, 3.7)	2.8 (1.7, 4.5)	< 0.001
GS area (cm^2^), median (IQR)	3.6 (1.8, 7.2)	4.1 (2.1, 8.1)	5.0 (3.1, 9.7)	9.5 (4.2, 17.6)	11.9 (4.8, 23.8)	< 0.001
Remnant myometrial thickness, (cm), median (IQR)	0.3 (0.3, 0.5)	0.3 (0.2, 0.5)	0.3 (0.2, 0.4)	0.2 (0.1, 0.2)	0.1 (0.0, 0.2)	< 0.001
Color doppler signal						0.024
No	44 (17.5)	50 (25.3)	11 (24.4)	13 (14.3)	24 (16.6)	
Yes	168 (66.9)	112 (56.6)	30 (66.7)	68 (74.7)	107 (73.8)	
Unknown	39 (15.5)	36 (18.2)	4 (8.9)	10 (11.0)	14 (9.7)	
Fetal heartbeat						0.18
No	127 (50.6)	104 (52.5)	25 (55.6)	61 (67.0)	83 (57.2)	
Yes	113 (45.0%)	87 (43.9)	18 (40.0)	24 (26.4)	56 (38.6)	
Unknown	11 (4.4%)	7 (3.5)	2 (4.4)	6 (6.6)	6 (4.1)	
Vaginal bleeding						< 0.001
No	87 (34.7)	71 (35.9)	9 (20.0)	20 (22.0)	26 (17.9)	
Yes	164 (65.3)	127 (64.1)	36 (80.0)	71 (78.0)	119 (82.1)	
Abdominal pain						0.23
No	187 (74.5)	150 (75.8)	28 (62.2)	63 (69.2)	99 (68.3)	
Yes	64 (25.5)	48 (24.2%)	17 (37.8)	28 (30.8)	46 (31.7)	
Preoperative hemoglobin, median (IQR)	126.0 (118.0, 131.0)	126.0 (117.0, 133.0)	124.0 (116.0, 129.0)	116.0 (102.0, 129.0)	115.5 (99.0, 124.5)	< 0.001
Preoperative HCG (mIU/ml), median (IQR)	29439.0 (9071.5, 56865.0)	38577.5 (12362.5, 72173.5)	30215.0 (5913.0, 57022.7)	24538.0 (3083.7, 60903.0)	26630.0 (2863.0, 65509.0)	0.069

Group 1, D&E under ultrasound; Group 2, D&E under hysteroscopy; Group 3, D&E under laparoscopy; Group 4, laparoscopy; Group 5, laparotomy; IQR, interquartile range; HCG, human chorionic gonadotrophin; CS, cesarean sections

### Comparison of the common complications and main outcomes among the five groups

There was no statistically significant difference observed in the occurrence of postoperative residual tissue among the five groups of patients (*p* = 0.053). Consequently, our clinical experience supports the adoption of the corresponding surgical approach. The occurrence of tissue residue was minimal across all patients, with no discernible variation among the five patient groups. (Table 2). However, significant differences were observed in intraoperative blood loss (> 200 ml), hemoglobin decline, first hemoglobin after operation, total hospital stay, hospital stay after operation, and transfusion among the five groups (*p* < 0.001). It should be noted that due to incomplete surgical records, there is a portion of cases where the intraoperative blood loss is unknown. In contrast, the HCG values did not exhibit any variation among the groups prior to surgery, as evidenced by the data presented in Table 1. However, a disparity in HCG values emerged after the second day following the surgical procedure. Furthermore, our analysis revealed no disparity in the occurrence of postoperative pain across the five patient groups (*p* = 0.72).

**Table 2:** Comparison of intraoperative bleeding occurrence, intraoperative complication occurrence, and main outcomes among the five groups

Factors	Group 1	Group 2	Group 3	Group 4	Group 5	*p* value
Number of patients	251 (%)	198 (%)	45 (%)	91 (%)	145 (%)	
Residual tissue						0.053
No	224 (89.2)	168 (84.8)	39 (86.7)	79 (86.8)	125 (86.2)	
Yes	10 (4.0)	20 (10.1)	3 (6.7)	2 (2.2)	6 (4.1)	
Unknown	17 (6.8)	10 (5.1)	3 (6.7)	10 (11.0)	14 (9.7)	
Intraoperative blood loss (mL)	75.6 (128.6)	66.6 (81.4)	91.7 (108.4)	171.4 (195.9)	305.5 (209.1)	< 0.001
Hemoglobin decline, median (IQR)	-10.6 (-17.0, -6.2)	-8.7 (-14.4, -5.4)	-10.8 (-16.5, -5.8)	-14.4 (-21.8, -6.8)	-15.7 (-21.7, -10.1)	< 0.001
First hemoglobin after operation, median (IQR)	110.0 (100.0, 118.0)	113.0 (103.0, 121.0)	111.0 (98.0, 115.0)	99.0 (89.7, 106.0)	94.0 (85.0, 105.0)	< 0.001
First HCG level after operation, median (IQR)	2727.0 (1073.0, 6320.0)	3487.5 (702.3, 7600.0)	2367.0 (748.7, 6635.3)	2377.0 (369.9, 5438.0)	1902.0 (244.3, 4791.0)	0.011
Total hospital stay (d), median (IQR)	8.0 (6.0, 10.0)	8.0 (5.5, 9.0)	9.5 (7.5, 13.5)	10.0 (8.0, 12.0)	11.0 (9.0, 13.0)	< 0.001
Hospital stay after operation (d), median (IQR)	5.0 (3.0, 6.0)	4.0 (3.0, 5.0)	6.0 (4.5, 7.0)	6.0 (6.0, 7.0)	7.0 (7.0, 8.0)	< 0.001
Pain						0.72
No	245 (97.6)	193 (97.5)	43 (95.6)	89 (97.8)	144 (99.3)	
Yes	2 (0.8)	1 (0.5)	1 (2.2)	0 (0.0)	1 (0.7)	
Unknown	4 (1.6)	4 (2.0)	1 (2.2)	2 (2.2)	0 (0.0)	
Transfusion						< 0.001
No	237 (94.4)	189 (95.5)	43 (95.6)	76 (83.5)	111 (76.6)	
Yes	9 (3.6)	6 (3.0)	1 (2.2)	14 (15.4)	33 (22.8)	
Unknown	5 (2.0)	3 (1.5)	1 (2.2)	1 (1.1)	1 (0.7)	

Group 1, D&E under ultrasound; Group 2, D&E under hysteroscopy; Group 3, D&E under laparoscopy; Group 4, laparoscopy; Group 5, laparotomy; IQR, interquartile range; HCG, human chorionic gonadotrophin

### Comparison of clinical outcomes between the MTX group and the non-MTX group

The surgical methods employed in the MTX group and the non-MTX group did not exhibit any differences (*p* = 0.02) **(**Table 3**)**. Similarly, there were no distinctions observed in the perioperative phase with regards to hemoglobin levels, including hemoglobin decline and the initial hemoglobin levels after the operation, between the two groups (*p* = 0.81 and 0.098). Patients in the MTX group exhibited a significantly higher amount of intraoperative blood loss compared to those in the non-MTX group (*p* < 0.001). It is noteworthy that the initial HCG level following the operation was higher in the MTX group than in the non-MTX group. However, there was no discernible pattern in HCG decline upon discharge. The requirement for perioperative transfusion did not differ between the two groups. Additionally, the MTX group experienced a more pronounced impact on hospital stay after the operation, total hospitalization duration, pain perception, and liver damage. The success rate of the operation, as indicated by residual tissue and reoperation, was found to be similar between the MTX and non-MTX groups (*p* = 0.086 and 0.17, respectively).

**Table 3:** Comparison of clinical outcomes between the MTX group and the non-MTX group

Factor	MTX group	Non MTX group	*p* value
Number of patients	283	297	
Operation methods			0.20
D&E under ultrasound	87 (30.7%)	108 (36.4%)	
Hysteroscopy	75 (26.5%)	85 (28.6%)	
D&E under laparoscopy	24 (8.5%)	15 (5.1%)	
Laparoscopy	41 (14.5%)	31 (10.4%)	
Laparotomy	56 (19.8%)	58 (19.5%)	
Intraoperative blood loss (ml), median (IQR)	50.0 (50.0, 100.0)	50.0 (20.0, 100.0)	< 0.001
Hemoglobin decline, median (IQR)	-12.0 (-17.7, -6.0)	-12.0 (-18.3, -6.2)	0.81
First hemoglobin after operation, median (IQR)	106.0 (96.0, 115.0)	109.0 (97.0, 118.0)	0.098
First HCG level after operation, median (IQR)	3453.0 (1515.0, 7295.0)	2394.0 (380.9, 5304.0)	< 0.001
HCG decline, median (IQR)	-90.7 (-94.0, -84.7)	-90.9 (-93.8, -86.4)	0.30
Transfusion	23 (8.1%)	21 (7.1%)	0.63
Liver damage	14 (4.9%)	2 (0.7%)	0.002
Residual tissue	22 (7.8%)	13 (4.4%)	0.086
Reoperation	21 (7.4%)	14 (4.7%)	0.17
Total hospital stay (d), median (IQR)	10.0 (8.0, 13.0)	8.0 (6.0, 9.0)	< 0.001
Hospital stay after operation (d), median (IQR)	6.0 (4.0, 7.0)	5.0 (4.0, 7.0)	0.012
Pain	4 (1.4%)	0 (0.0%)	0.040

IQR, interquartile range; HCG, human chorionic gonadotrophin;

### An analysis of surgical procedure frequencies conducted using different classification methods

**Fig. 2:**
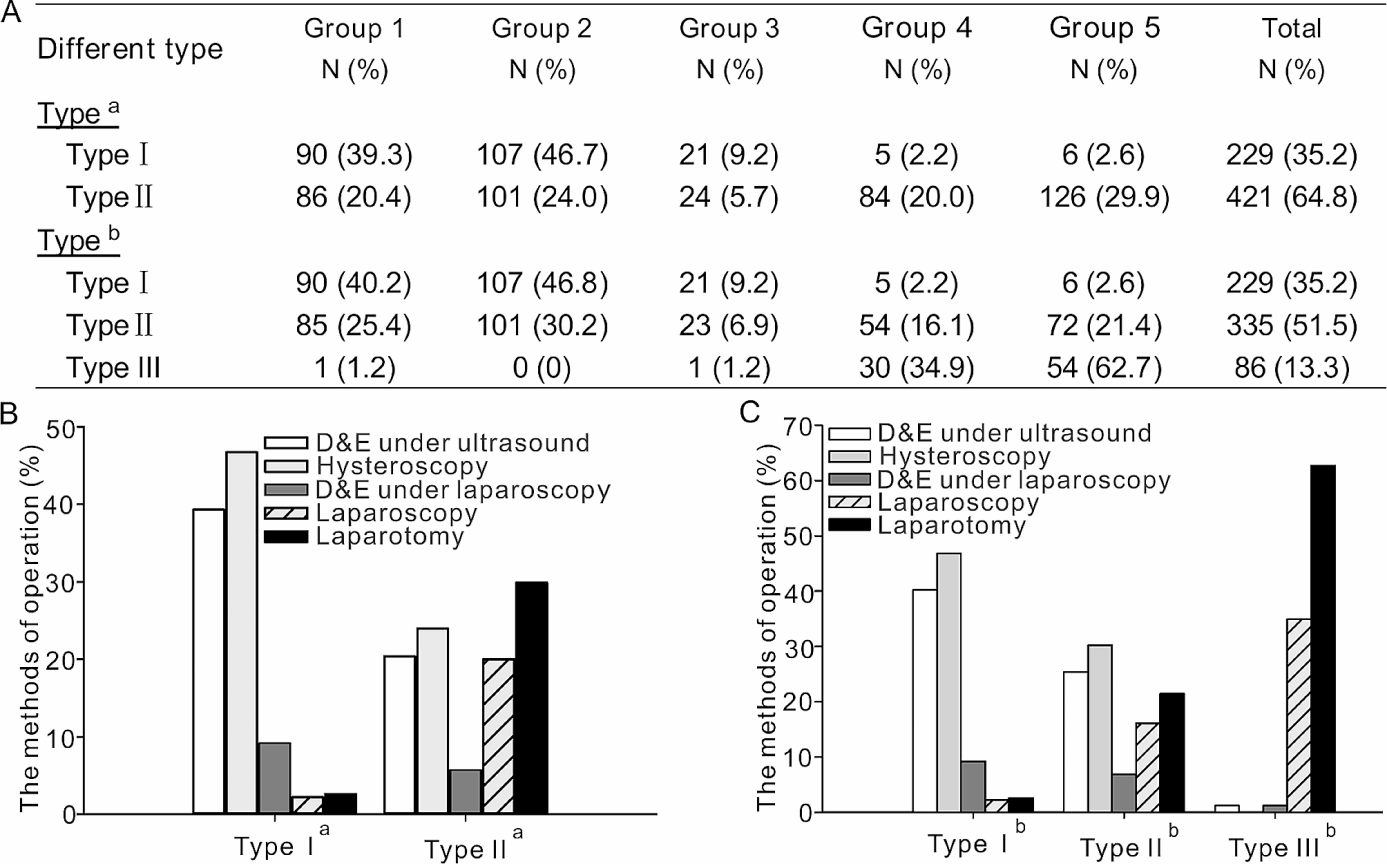
The method of operation according to the different types of CSP (%). The CSP cases were categorized based on two distinct classification methodologies. An examination was conducted to assess the variances in treatment approaches employed across these classification methods. (A) the quantification and proportion of cases falling under each treatment scheme within different classification methods; (B) the classification of patients with CSP according to the system proposed by Vial Y, with the accompanying bar chart illustrating the proportion of patients treated using various surgical methods; (C) a bar chart depicting the percentage of cases within different groups based on the clinical recommendations put forth by Chinese scholars

Based on the two different classification methods^[^, the CSP cases were typed accordingly. The study examined the treatment methods employed in the two classification systems. Within the binary classification system, a significant proportion of type I^a^ CSP patients received group 1 and group 2 treatments (39.3% and 46.7% respectively), while approximately half of the type II^a^ CSP patients underwent group 4 and group 5 treatments (20.0% and 29.9% respectively) **(**Fig. 2A and B**)**. In the three-way classification system, group 1 and group 2 continued to predominantly consist of type I^b^ CSP patients. Notably, 62.7% of patients in group 5 were classified as type III^b^ CSP, while 34.9% of patients in group 4 were also identified as type III^b^ CSP **(**Fig. 2A and C**)**. It was challenging to differentiate the proportions of type II^a^ and type II^b^ patients receiving the five treatment regimens using the two classification methods. Hence, the selection of suitable surgical techniques for patients with these types of CSP poses a challenge for medical practitioners.

### Analysis of the misdiagnosis of CSP

**Fig. 3:**
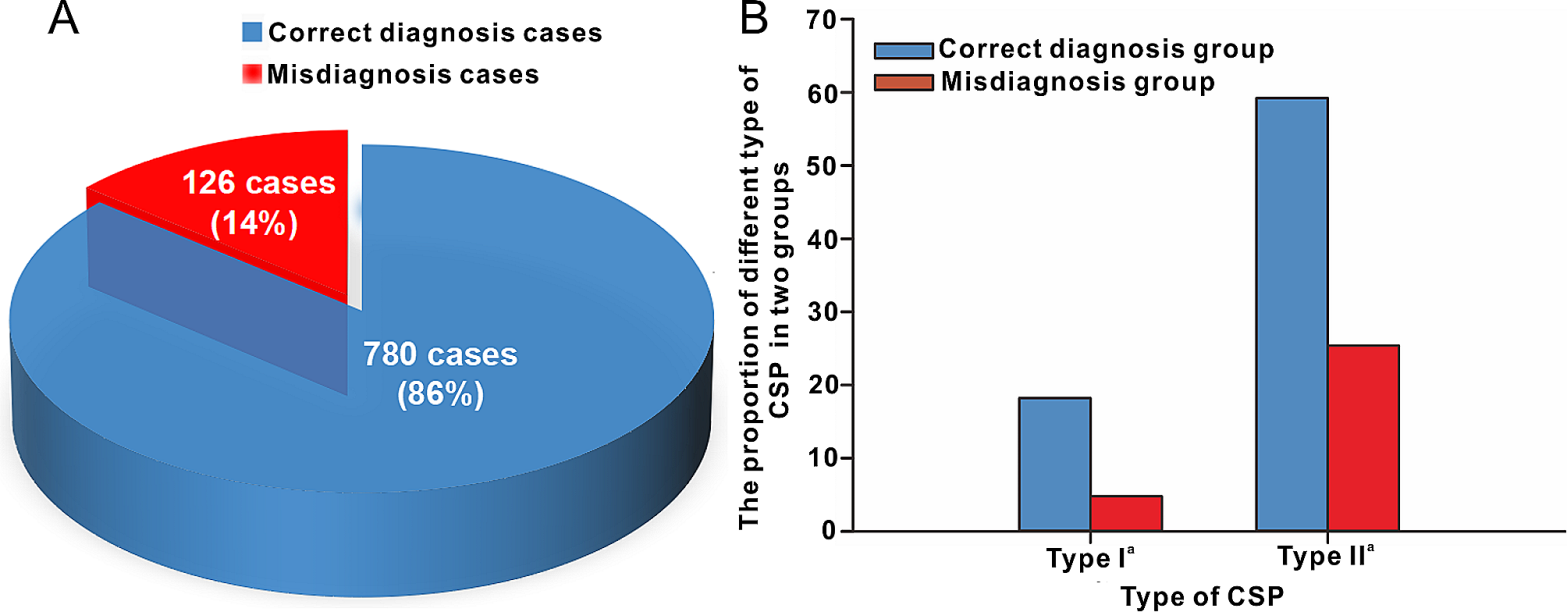
CSP patient misdiagnoses according to the different types of CSP. (A) Pie chart of the percentage of CSP patients misdiagnosed and correctly diagnosed. (B) The proportion of misdiagnosed cases and correctly diagnosed cases according to different types of CSP.

As a result of the diverse levels of risk linked to the erroneous diagnosis of CSP or the misidentification of CSP as a typical intrauterine pregnancy, patients may encounter varying levels of bleeding during the surgical termination of pregnancy. Consequently, we undertook a comprehensive comparative analysis encompassing all patients who were misdiagnosed and those who were accurately diagnosed. Among the 906 patients examined, 126 (14%) were erroneously diagnosed with a typical intrauterine pregnancy, while 780 (86%) were correctly diagnosed with CSP (Tables 4**and** Fig. 3A). Notably, this study revealed a higher incidence of misdiagnosis in cases of type II^a^ CSP compared to type I^a^ CSP (Tables 4**and** Fig. 3B).

**Table 4:** Comprehensive analysis of misdiagnosed cesarean scar pregnancy patients

Factor	Correct diagnosis group	Misdiagnosis group	*p* value
Number of patients	780	126	
Type			< 0.001
Type I^a^	142 (18.2%)	6 (4.8%)	
Type II^a^	462 (59.2%)	32 (25.4%)	
Number of artificial abortions, median (IQR)	2.0 (1.0, 3.0)	2.0 (1.0, 3.0)	0.054
Number of cesarean sections, median (IQR)	1.0 (1.0, 2.0)	1.0 (1.0, 2.0)	0.51
Interval from last CS (years), median (IQR)	5.0 (3.0, 8.0)	5.0 (3.0, 8.0)	0.95
Gestational age (days), median (IQR)	49.0 (43.0, 58.0)	58.5 (49.0, 74.5)	< 0.001
Diameter of GS (cm), median (IQR)	2.9 (2.0, 4.1)	4.3 (3.2, 5.6)	< 0.001
GS width (cm), median (IQR)	1.5 (1.0, 2.4)	3.3 (2.1, 4.7)	< 0.001
GS area (cm^2^), median (IQR)	4.5 (2.2, 9.4)	14.9 (6.8, 25.2)	< 0.001
Remnant myometrial thickness (cm), median (IQR)	0.3 (0.2, 0.4)	0.1 (0.0, 0.2)	< 0.001
Color Doppler signal	502 (64.4%)	101 (80.2%)	< 0.001
Fetal heartbeat	358 (45.9%)	16 (12.7%)	< 0.001
Vaginal bleeding	522 (66.9%)	113 (89.7%)	< 0.001
Abdominal pain	221 (28.3%)	33 (26.2%)	0.62
Preoperative hemoglobin, median (IQR)	124.0 (116.0, 131.0)	110.0 (93.0, 119.0)	< 0.001
Preoperative HCG (mIU/ml), median (IQR)	35236.5 (11620.0, 71043.5)	2702.0 (407.4, 18631.0)	< 0.001
Treatments methods			< 0.001
D&E with ultrasonographic guidance	239 (38.3%)	12 (11.3%)	
Hysteroscopy	187 (30.0%)	11 (10.4%)	
D&E with laparoscopic guidance	37 (5.9%)	8 (7.5%)	
Laparoscopy	63 (10.1%)	28 (26.4%)	
Laparotomy	98 (15.7%)	47 (44.3%)	
Liver damage	18 (2.3%)	5 (4.0%)	0.27
Residual tissue	30 (3.8%)	14 (11.1%)	< 0.001
Reoperation	27 (3.5%)	38 (30.2%)	< 0.001
Intraoperative blood loss (ml), median (IQR)	50.0 (30.0, 100.0)	100.0 (50.0, 200.0)	< 0.001
First hemoglobin after operation, median (IQR)	109.0 (98.0, 117.0)	96.0 (86.0, 106.0)	< 0.001
First HCG level after operation, median (IQR)	3097.0 (1018.0, 6978.0)	414.4 (39.0, 2000.2)	< 0.001
Hemoglobin decline, median (IQR)	-11.4 (-17.8, -6.3)	-12.0 (-18.6, -5.7)	0.63
hCG decline, median (IQR)	-90.7 (-93.9, -84.8)	-91.7 (-94.5, -80.1)	0.73
Total hospital stay (d), median (IQR)	8.0 (7.0, 11.0)	10.0 (8.0, 12.5)	< 0.001
Hospital stay after operation (d), median (IQR)	5.0 (4.0, 7.0)	7.0 (6.0, 8.0)	< 0.001
Pain	5 (0.6%)	2 (1.6%)	0.26
Transfusion	46 (5.9%)	30 (23.8%)	< 0.001

Group 1, D&E under ultrasound; Group 2, D&E under hysteroscopy; Group 3, D&E under laparoscopy; Group 4, laparoscopy; Group 5, laparotomy; IQR, interquartile range; HCG, human chorionic gonadotrophin; CS, cesarean sections

Due to the occurrence of intrauterine procedures in other medical facilities for misdiagnosed patients, the determination of the stage of CSP prior to initial treatment remains uncertain. The two groups of patients exhibited no discernible disparities in terms of pregnancy history, encompassing the number of artificial abortions, cesarean sections, or the duration since the most recent cesarean section. The study revealed substantial variations in various preoperative indicators (including gestational age, diameter of the GS, GS width, GS volume, remnant myometrial thickness, color Doppler signal, fetal heartbeat, and vaginal bleeding) as well as perioperative factors (such as preoperative hemoglobin levels, treatment methods, presence of residual tissue, need for reoperation, intraoperative blood loss, first hemoglobin levels post-operation, first HCG levels post-operation, total hospital stay, post-operative hospital stay, and transfusion requirements) (*p* < 0.001). However, there were no statistically significant variations noted in the two indicators (hemoglobin decline and HCG decline).

## Discussion

CSP is a multifaceted iatrogenic disorder associated with pregnancy, potentially resulting in severe complications throughout gestation. Currently, the prevalence of CSP is on the rise due to the escalating rates of cesarean section procedures. However, the most effective treatment for CSP remains uncertain, as it should ideally exhibit a high success rate, minimal complications, and, whenever feasible, preserve the patient’s fertility. The objective of this study is to comprehensively assess the effectiveness of various treatment approaches for CSP. Our research indicates that D&E under ultrasound or D&E under hysteroscopy is a suitable treatment option for patients diagnosed with type I CSP. On the other hand, operative resection (laparoscopy or laparotomy) should be employed for patients with type III^b^ CSP. However, determining the most appropriate method for treating type II CSP poses a significant challenge.

### Conservative management

Numerous studies have indicated that expectant management may be a feasible approach for early non-viable cervical ectopic pregnancies. Patients in this category should receive regular monitoring of symptoms, HCG levels, ultrasound signs, and demonstrate favorable clinical outcomes [14]. However, the occurrence of serious bleeding and uterine arteriovenous malformation is possible when employing expectant management, thus leading the Society for Maternal-Fetal Medicine to not recommend this approach [15]. Due to the potential for maternal morbidity, many doctors advocate for the early termination of CSP. Furthermore, when CSP is accompanied by an irregularly shaped cesarean scar diverticulum (CSD), treatment becomes more complex, increasing the likelihood of residual tissue or scar rupture, resulting in bleeding and potential damage to the bladder and uterine artery. Consequently, the investigation of a novel and appropriate classification system for CSP holds immense significance in mitigating the aforementioned complications.

### Methotrexate management

The use of MTX treatment for CSP has been recommended by some doctors and associations as an effective method of treatment, as it has been shown to reduce the rate at which HCG levels increase. A meta-analysis study demonstrated the ability of MTX to lower HCG levels in the treatment of CSP [16]. Furthermore, a review study suggested that local injection of MTX can be considered as a first-line treatment option [17]. In our own study, we observed no significant differences in surgical methods between the MTX and non-MTX groups (*p* = 0.02). However, it is important to note that CSP patients in the MTX group experienced significantly higher levels of intraoperative blood loss compared to those in the non-MTX group. Simultaneously, the initial postoperative HCG level in the MTX group exceeded that of the non-MTX group, thereby undermining our rationale for employing MTX as a therapeutic intervention for CSP. Nevertheless, no disparity was observed in the pattern of HCG reduction post-operation between the MTX and non-MTX groups.

### Dilatation and curettage

D&E performed under ultrasound guidance has been extensively employed for the management of CSP, either as a standalone procedure or in conjunction with adjuvant medical therapy. While certain studies have reported successful outcomes with D&E as a treatment for CSP, alternative perspectives have deemed it ineffective and yielding insufficient results [18]. The utilization of ultrasound during D&E procedures has notably enhanced physicians’ ability to visualize the GS and cesarean scar, consequently reducing the likelihood of complications and endometrial injury [19]. According to Huo’s study [6], it was demonstrated that D&E was deemed safe for patients with type I^b^ CSP, regardless of the size of the GS. However, it was found to be inadequate and hazardous for patients with type II^b^ CSP and particularly perilous for those with type III^b^ CSP. Nevertheless, there are also some doctors who posit that arteriovenous malformation may arise subsequent to D&E [20]. In our own investigation, we similarly discovered that a majority of type I^b^ CSP patients underwent either ultrasound-guided D&E (40.2%) or hysteroscopy-assisted D&E (46.8%), which was associated with reduced intraoperative blood loss and a shorter duration of hospitalization. Therefore, in the case of stable type I CSP patients, the utilization of D&E guided by ultrasound or hysteroscopy is deemed appropriate.

### Hysteroscopy

Additionally, some researchers have advocated for hysteroscopy as a favorable alternative for patients with type I^a^ CSP [21]. Hysteroscopy provides the benefit of direct visualization and is associated with a high rate of success and minimal complications in the management of CSP [22]. Multiple studies [23] have demonstrated that complications related to hysteroscopy are rare. A comprehensive review conducted by Sarah Maheux-Lacroix et al. encompassed 63 studies and examined diverse treatment modalities for CSP [24]. In their investigation, the researchers found that the use of D&E treatment alone was associated with a 28% likelihood of hemorrhage. However, when combined with UAE, this risk decreased to 4%. In our own cases, a total of 28 patients underwent UAE alongside other vascular management measures, and no significant disparity in intraoperative bleeding was observed between these patients and those who did not undergo UAE (*P* > 0.05) [13]. However, Salmeri et al. indicated that UAE can effectively manage postpartum hemorrhage involving the uterine arteriovenous malformations [25]. The potential impact of UAE on fertility has consistently been a matter of concern. Nevertheless, a recent investigation proposes that UAE did not exert a significant influence on ovarian function [26]. Furthermore, the employment of hysteroscopy treatment was associated with a failure rate of 12%. It is worth noting that if the shape of the niche is irregular, both of the aforementioned methods are more likely to result in the presence of residual tissue in the corners of the niche.

### Operative resection

For type II^a^ and type III^b^ CSP, operative resection is deemed appropriate. Numerous studies have demonstrated the efficacy of laparoscopic hysterotomy with wedge resection of the previous scar, thus warranting its recommendation [27, 28]. In a review study, laparoscopy demonstrated a success rate of 97% with expedited resolution of HCG levels and absence of complications [22]. However, in the present study, over half of type III^b^ patients (62.7%) opted for laparotomy. Our previous study compared the outcomes of laparotomy and laparoscopy in the treatment of CSP. Patients with CSP who underwent laparoscopy experienced a shorter hospital stay, reduced postoperative hospital length of stay, decreased intraoperative bleeding, and fewer blood transfusions [13]. Further evaluation is necessary to determine the potential benefits of excision and repair of scar defects on subsequent pregnancy outcomes. Differentiating between the four schemes in type II^a^ and type II^b^ CSP proved challenging in this study. The selection of an optimal treatment strategy for type II CSP is a pressing concern for gynecologists. Discriminating between type III^a^ and type III^b^, which exhibit similar scar thickness but differ in the protrusion of the GS and presence of a 0.3 cm muscular layer, remains uncertain.

Our investigation also shed light on an additional critical facet pertaining to the misdiagnosis of CSP, an issue that has not garnered substantial attention from doctors. The existing literature solely comprises sporadic case reports [29, 30]. Following the occurrence of amenorrhea, doctors commonly employ ultrasound as a crucial diagnostic tool to ascertain the presence of pregnancy, thereby attributing significant importance to ultrasound in the diagnosis of CSP. The clinical manifestations of CSP lack specificity, often resulting in doctors disregarding its significance during the initial stages of pregnancy within clinical settings. The potential misdiagnosis of CSP can consequently yield erroneous treatment approaches and pose a grave threat to patients’ lives due to the potential occurrence of severe hemorrhage. In our study, a notable proportion (14%) of patients diagnosed with CSP were found to have been misdiagnosed with intrauterine pregnancy. These misdiagnosed patients exhibited a longer gestational age in comparison to those who were correctly diagnosed (58.5 days vs. 49.0 days). Consequently, these individuals encountered varying degrees of intraoperative and prolonged postoperative vaginal bleeding during the termination of the pregnancy. Thus, the timely and accurate identification of CSP can effectively prevent misdiagnoses, which have the potential to result in the development of placenta accreta spectrum (PAS) [31] or other severe life-threatening complications.

The present study conducted a comprehensive evaluation of the prevailing treatments for CSP, yielding specific evidence for clinicians. Nevertheless, several limitations were identified. Firstly, the study design was retrospective and confined to a single center, potentially introducing selection bias. Secondly, the inclusion of treatment strategies chosen by different physicians may have introduced bias stemming from variations in their experiences. Lastly, the absence of a long-term follow-up in our study represents another limitation.

In summary, the primary treatment option for patients with type I CSP is dilation and evacuation (D&E) performed under ultrasound guidance or hysteroscopy, while operative resection is recommended for patients with type IIIb CSP. It is currently challenging to determine the suitable treatment methods for patients diagnosed with type II^a^ or II^b^ CSP. To address this issue, further research is required to investigate a novel classification method for CSP. To determine the most effective treatment strategy, it is essential to expand retrospective sample sizes, incorporate data from multiple centers, and conduct randomized controlled trials (RCTs) to thoroughly assess the advantages, disadvantages, and economic considerations of both treatment modalities. Enhancing the early first-trimester diagnosis of CSP is essential to prevent misdiagnoses.

## Data Availability

The datasets generated during the current study are not publicly available.
